# Effectiveness of Selected Fitness Exercises on Stress of Femoral Neck using Musculoskeletal Dynamics Simulations and Finite Element Model

**DOI:** 10.2478/hukin-2014-0033

**Published:** 2014-07-08

**Authors:** Jing-Guang Qian, Zhaoxia Li, Hong Zhang, Rong Bian, Songning Zhang

**Affiliations:** 1 Department of Sport Science, Nanjing Sport Institute, Nanjing, China.; 2 Department of Engineering Mechanics, Southeast University, Nanjing, China.; 3 Biomechanics/Sports Medicine Lab, The University of Tennessee, Knoxville, TN.

**Keywords:** femoral neck fracture, finite-element model, LifeMOD, exercise, compression, tension

## Abstract

The purpose of the study was to establish a dynamics model and a three-dimensional (3D) finite element model to analyze loading characteristics of femoral neck during walking, squat, single-leg standing, and forward and lateral lunges. One male volunteer performed three trials of the five movements. The 3D kinematic data were captured and imported into the LifeMOD to establish a musculoskeletal dynamics model to obtain joint reaction and muscle forces of iliacus, gluteus medius, gluteus maximus, psoas major and adductor magnus. The loading data LfeMOD were imported and transformed into a hip finite-element model. The results of the finite element femur model showed that stress was localized along the compression arc and the tension arc. In addition, the trabecular bone and tension lines of the Ward’s triangle also demonstrated high stress. The compact bone received the greatest peak stress in the forward lunge and the least stress in the squat. However, the spongy bone in the femoral neck region had the greatest stress during the walk and the least stress in the squat. The results from this study indicate that the forward lunge may be an effective method to prevent femoral neck fractures. Walking is another effective and simple method that may improve bone mass of the Ward’s triangle and prevent osteoporosis and femoral neck fracture.

## Introduction

The age of elderly population has gradually increased in the 21th century (WHO, 2002). It was estimated that the elderly population reached 88.11 million, accounting for approximately 6.96% of the entire population in China in 2000, and this number further increased to 130 million in 2003 accounting for 10% of population in China (Guo et al., 2001). Furthermore, it was speculated that the elderly population would achieve 160 million or 12% of the entire population by 2010. The increasing rate of aging population suggests that China has started to enter into a most rapid development period of aging (Guo et al., 2001; Li et al., 2001). Osteoporosis has drawn much attention in research in the past two decades. World Health Organization (WHO) has designated this past decade as the “The Bone and Joint Decade 2000–2010” (Brundtland, 2000; Lidgren, 2003; Zhang and Pei, 2003). Treatment and prevention of osteoporosis, femoral neck fractures, and femoral head necrosis disease have become focal points of medical research in gerontology.

Femoral neck fracture is the most serious osteoporotic fracture of human body and has the highest prevalence rate. As early as in 1824, Cooper suggested a significant decrease of bone mineral density (BMD) at the site between femoral head and neck among osteoporosis patients which may lead to occurrence of femoral neck fracture (Wang, 2002). The prevalence rate of femoral neck fracture in the elderly population is 68.4% among all hip fractures and increases 40% for every decade of age. It was reported that the rate of necrosis after femoral neck fracture was approximately 20 – 40% (Liu et al., 2005). Common consequences such as difficulty of bone coalescence and necrosis of femoral head have remained challenging in current medical treatments (Estrada et al., 2002). Therefore, early prevention against the fracture has been considered as an important measure according to the findings from many clinical studies (Chen et al., 2001).

In order to investigate influences of exercises on femoral neck, computer simulations of human musculoskeletal systems and movement dynamics are necessary in order to examine stress distributions across the femoral neck. There are several computer simulation packages that are designed specifically for human dynamics and musculoskeletal simulations. SIMM is a widely used software system for modeling the musculoskeletal system (Delp et al., 1990). OpenSim is an open-source software system that lets users create and analyze dynamic simulations of movement (Delp et al., 2007), which is being developed at Simbios, a National Institute of Health center at Stanford University for physics-based simulation of biological structures. LifeMOD and AnyBody (Taylor et al., 2011) are also specialized in simulations of musculoskeletal simulations of human movements.

Our group has conducted several studies to explore the mechanisms of femoral neck fractures. We have previously shown that the cause of femoral neck fracture is related to both bone mineral density and femoral neck structure (Qian et al., 2009). Patients with femoral neck fractures exhibited a smaller femoral neck angle and lower bone mineral density in the Ward’s triangle area compared to healthy population. It was also found that the stress of the fracture patients can reach the ultimate strength level rather easily which may lead to fractures under same loading condition. Especially when the femoral neck angle of one side was significantly less than the other side and less than 125°, the risk of femoral neck fracture increased significantly. It was highly recommended that prevention measures such as targeted strength training and improved self-awareness of falls and impacts should be emphasized in order to reduce possible injuries and accidents.

To study fracture risks associated with several sites of femoral neck during human movements, we adopted a modeling approach of a hip joint (Qian and Bian, 2009). Pelvis and bilateral femurs were modeled in a finite element model based upon Dicom CT images with different material properties applied to acquire descriptions of non-uniform material properties of the bone tissue. The relationships among stress and strain and bone mineral density at 12 monitoring points of the femur in a standing phase were also investigated with loading of body gravity.

Therefore, the purpose of the study was to establish a dynamics model and a three-dimensional (3D) finite element model to analyze loading characteristics of femoral neck during walking, squat, single-leg standing, and forward and lateral lunges. A human body is a great and self-modifying system. When stresses applied to osseous structures are within physiological ranges, the balance between the activities of osteoclasts and osteoblasts can change to decrease osteoclast activities and increase osteoblast activities in order to increase bone mass, bone mineral density and bone strength (Cowin, 1993). Through biomechanical investigation of daily fitness exercises, we hoped to find out which exercises can produce proper stress to femur in order to provide useful information on exercises designed for prevention of femoral neck fracture and reduction of injury rates.

## Material and Methods

### Motion capture

A male volunteer participated in the study (age: 23 year, body height: 1.70 m, body mass: 60 kg) and provided a written informed consent approved by the local ethics committee. He was asked to perform three trials of five different movements: level walking, squat, one leg standing, forward lunge and lateral lunge. Thirty one reflective markers were placed on the head, cervical and thoracic spine, clavicles, sternum, and both sides of upper and lower extremities. The 3D kinematic data of the movements were captured using a 3D motion capture system (60 Hz, Motion Analysis System, USA). The data from one successful trial of each movement was used for further analyses.

### LifeMOD Musculoskeletal Dynamics Modeling

**Figure 1:**
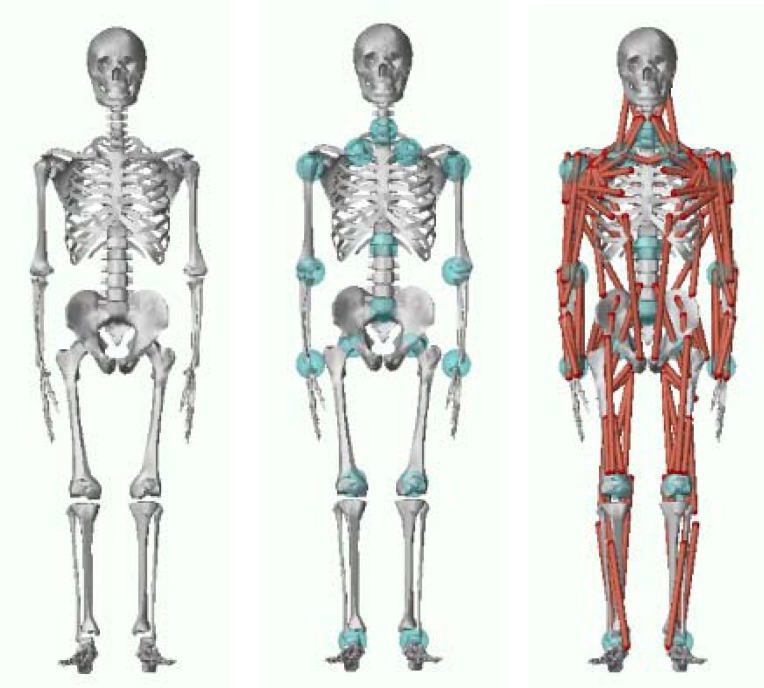
A) Human skeletal model, B) skeletal model with joints, and C) musculoskeletal model.

The anthropometric parameters of the participant including body height and mass, and circumferences of body segments were measured and used in modeling in LifeMOD (LifeModeler, Inc., San Clemente, CA). The collected 3D kinematic data were exported to the LifeMOD. The modeling of body segments and analysis of data were performed in a series of steps. First, a customized skeleton model was established based on the participant’s height, mass, gender, age as well as a series of anthropometric measurements (Figure 1A). Kinematic joints were then defined to connect body segments and equations of degrees of freedom for each joint were established. Kinematic joints were set to be spring-damping units to stabilize the model during dynamics simulation (Figure 1B). A total of 118 muscles was then created which were attached to bones at relevant anatomical landmarks (Figure 1C). Kinematic trajectories from the collected kinematics were then prescribed to the model to drive and control its motion. An equilibrium analysis was performed and potential energy was minimized to reduce discrepancy between the model and the prescribed motion. A foot-ground contact was then created using a ground model to simulate the contact between the human body and ground. Through an inverse dynamics analysis, discrepancies between collected motion trajectories and simulated trajectories of the model during the motion were recorded and used later in forward dynamics analysis. Finally, joint reaction forces and muscle forces were computed through the forward dynamic simulation. Muscle forces of the iliacus, gluteus medius, gluteus maximus 1 (upper fibers), gluteus maximus 2 (lower fibers), adduct magnus and psoas major were selected for further analyses.

To verify the reliability of joint reaction forces and muscle forces generated in LifeMOD, results from previous studies were compared with those from our research. Some of previous studies have focused on the joint reaction forces and ignored contributions from muscle forces. The peak hip joint contact force during normal walking was found to be 2.18 times of body weight (BW) (Zheng, 2007). In this study, the peak hip joint contact force was 1308N resulting a peak knee joint reaction force of 2.22 BW, which is comparable to the peak hip joint contact force found in the previous study (Zheng, 2007).

LIFEMOD was used to simulate the five different movements. Inverse dynamic simulation and calculation were applied to obtain joint and muscle force of the involved bone during movement. According to the symmetry of the femur, left femur was selected for joint and muscle forces analysis.

### Hip finite element model and transformation of two different coordinate systems

The hip finite element model with four unique material properties was established based upon a previous study (Qian and Bian, 2009). The joint contact and muscle forces computed through the forward dynamics simulation in LIEFMODE cannot be used directly in the hip model established in ANSYS (9.0, Canonsburg, Pennsylvania) due to the differences in the coordinate systems used in LifeMOD and the finite element software. Therefore, the transformation of coordinates from LifeMOD to ANASYS was necessary and performed through a customized Matlab program.

Two coordinate systems were created: *σ* = [*O*;*e*_1_, *e*_2_, *e*_3_] and *σ*′ = [*O*′;*e*_1_′, *e*_2_′, *e*_3_′]. The symbol *σ* is designated as the original coordinate system in LIEFMOD while *σ*′ is for the new coordinate system in ANASYS. *e*_1_, *e*_2_, *e*_3_, the orthogonal unit vectors of *σ*, were written as the linear combination of *e*_1_′, *e*_2_′, *e*_3_′.

(1) $$\begin{array}{l} e_{1}=b_{11}{e_{1}}^{\prime }+b_{12}{e_{2}}^{\prime }+b_{13}{e_{3}}^{\prime }\\ e_{2}=b_{21}{e_{1}}^{\prime }+b_{22}{e_{2}}^{\prime }+b_{23}{e_{3}}^{\prime }\\ e_{3}=b_{31}{e_{1}}^{\prime }+b_{32}{e_{2}}^{\prime }+b_{33}{e_{3}}^{\prime } \end{array}$$

**Figure 2:**
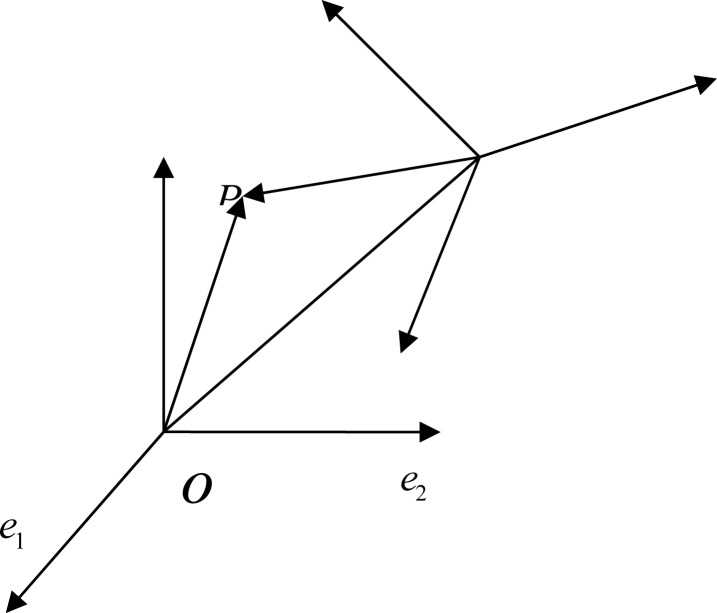
Coordinate transformation between the original coordinate system σ in LIEFMOD and the new coordinate system σ′ in ANASYS.

P is designated a point in the space, and its coordinates in *σ* and *σ*′ were (*x,y,z*) and (*x*′*,y*′*,z*′), respectively. The coordinate of *O*′ in *σ*′ was *x*_0_, *y*_0_, *z*_0_ (Figure 2). The components of $\vec{O^{\prime }P}=\vec{O^{\prime }O}+\vec{OP}$ in *σ* and *σ*′ were (*x* − *x*_0_, *y* − *y*_0_, *z* − *z*_0_) and (*x*′, *y*′, *z*′), so

(2) $$\vec{O^{\prime }P}=\left(x-x_{0}\right)e_{1}+\left(y-y_{0}\right)e_{2}+\left(z-z_{0}\right)e_{3}$$

(3) $$\vec{O^{\prime }P}=x^{\prime }{e_{1}}^{\prime }+y^{\prime }{e_{2}}^{\prime }+z^{\prime }{e_{3}}^{\prime }$$

Place (1) into (2) and then compare it with (3), we can obtain the following.

(4) $$\{\begin{array}{l} x^{\prime }=b_{11}\left(x-x_{0}\right)+b_{21}\left(y-y_{0}\right)+b_{31}\left(z-z_{0}\right)\\ y^{\prime }=b_{12}\left(x-x_{0}\right)+b_{22}\left(y-y_{0}\right)+b_{32}\left(z-z_{0}\right)\\ z^{\prime }=b_{13}\left(x-x_{0}\right)+b_{23}\left(y-y_{0}\right)+b_{33}\left(z-z_{0}\right) \end{array}$$

or

(5) $$\{\begin{array}{l} x^{\prime }=b_{11}x+b_{21}y+b_{31}z+{x_{0}}^{\prime }\\ y^{\prime }=b_{12}x+b_{22}y+b_{32}z+{y_{0}}^{\prime }\\ z^{\prime }=b_{13}x+b_{23}y+b_{33}z+{z_{0}}^{\prime } \end{array}$$

Thus,

(6) $$\begin{array}{l} \left(\begin{array}{c} x^{\prime }\\ y^{\prime }\\ z^{\prime }\\ 1 \end{array}\right)=\left(\begin{array}{cccc} b_{11} & b_{21} & b_{31} & {x_{0}}^{\prime }\\ b_{12} & b_{22} & b_{32} & {y_{0}}^{\prime }\\ b_{13} & b_{23} & b_{33} & {z_{0}}^{\prime }\\ 0 & 0 & 0 & 1 \end{array}\right)\left(\begin{array}{c} x\\ y\\ z\\ 1 \end{array}\right)\\ \{\begin{array}{l} {x^{\prime }}_{0}=-\left(b_{11}x_{0}+b_{21}y_{0}+b_{31}z_{0}\right)\\ {y^{\prime }}_{0}=-\left(b_{12}x_{0}+b_{22}y_{0}+b_{32}z_{0}\right)\\ {z^{\prime }}_{0}=-\left(b_{13}x_{0}+b_{23}y_{0}+b_{33}z_{0}\right) \end{array} \end{array}$$

Where equations 5 and 6 are referred as the affine coordinate transformation equations from *σ* to *σ′*.

### Data Analysis

Data of three critical events of the five tested movements, early stance, mid stance, and late stance, were selected for analysis because the joint reaction forces (JRF) and muscle forces at these critical times were typically at their maximum or minimum (Table 1).

## Results

### Loading to femur during different movements

**Figure 3:**
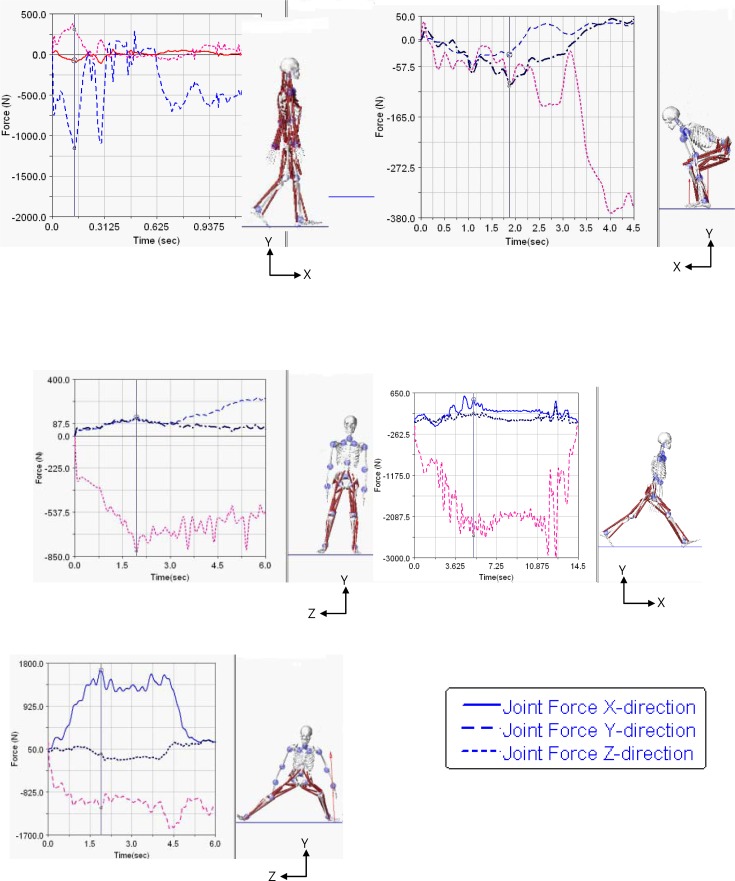
Joint reaction forces of five tested fitness movements. The three vertical lines on the curves represent three critical times, early, mid and late stages of a) walking, b) squat, c) one-leg standing, d) forward lunge, and e) lateral lunge.

The hip JRFs from LifeMod were analyzed at the three different times, at early (loading response), mid (mid-stance), and late stance (swing leg heel-strike), which represent maximum and minimum loading during level walking (Figure 3a). The results showed that the peak hip compressive force reached over 650 N and 390 N in the early and late stance (Table 1). Figure 3b shows the hip JRF patterns in the squat with the three vertical lines representing early (maximum knee flexion), mid (mid extension), and late stance (erect position). The compressive JRF in ANSYS showed smaller loading in the early and mid-stance, and higher loading in the late stance (Table 1). Figure 3c shows the hip JRF patterns of LifeMod in the one-leg standing with the 3 lines representing the early (swing leg leaving ground), mid (mid swing) and late stance (swing leg reaching highest point). The ANSYS compressive hip JRF reached similar levels of loading at the three different critical times (Table 1). The hip JRFs in LifeMod were provided in Figure 3d with values analyzed at early (touchdown of front leg), mid (lowest point of center of gravity (COG)), and late stance (trailing leg off ground). The compressive JRF in ANSYS was higher in the early and mid- stance while the shear JRF was highest in the late stance and lowest in the mid stance (Table 1). Figure 3e shows the JRF patterns of the lateral lunge with the 3 lines representing the early (leading leg touchdown), mid (lowest COG position), and late stance (contralateral leg off ground). The ANSYS compressive and shear forces were highest in late stance in the movement (Table 1).

### Femur neck loads and boundary conditions

**Figure 4:**
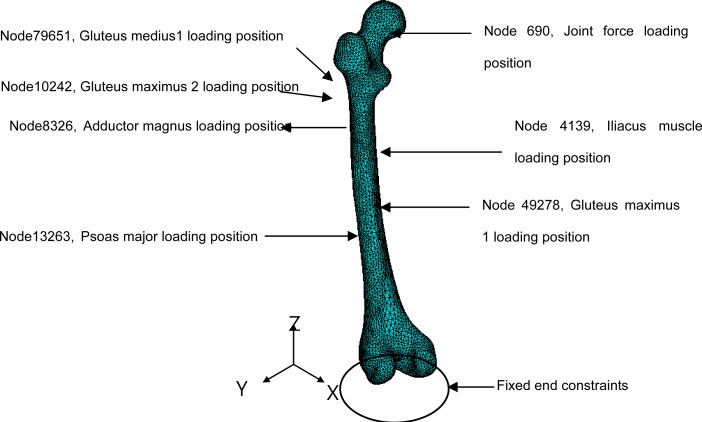
Illustrations of loading applications to the proximal femur.

After the distal end of the femur was constrained with zero degrees of freedom as the boundary conditions in the 3D finite element model of the femur, the joint reaction forces were applied to the center of the femoral head and the forces of the six muscles (Figure 4) were applied to the corresponding locations. The femoral head center was represented as the node 690 in ANSYS. The nodes representing the muscle loads were 4139 for the iliacus, 79651 for the gluteal muscle 1, 10242 for the gluteus maximus 2, 13236 for the psoas major, 49278 for the gluteus maximus, and 8326 for the adductor magnus (Figure 4).

### Stress distribution of femoral neck during different gaits

**Figure 5:**
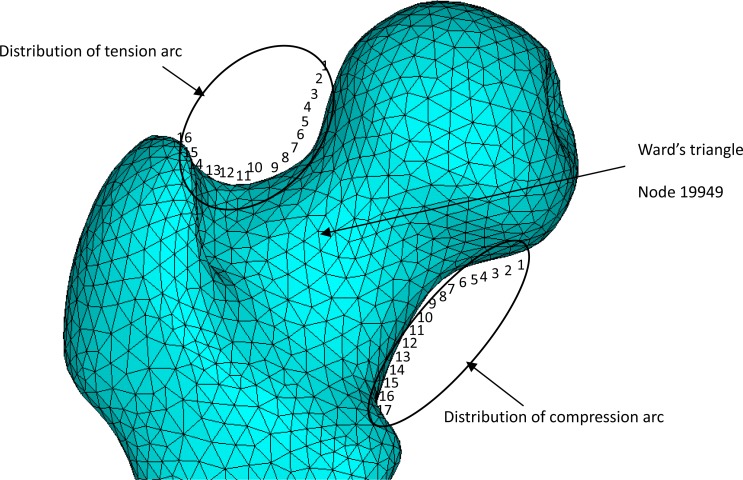
Stress location points around the femoral neck in the femur finite element model.

**Figure 6:**
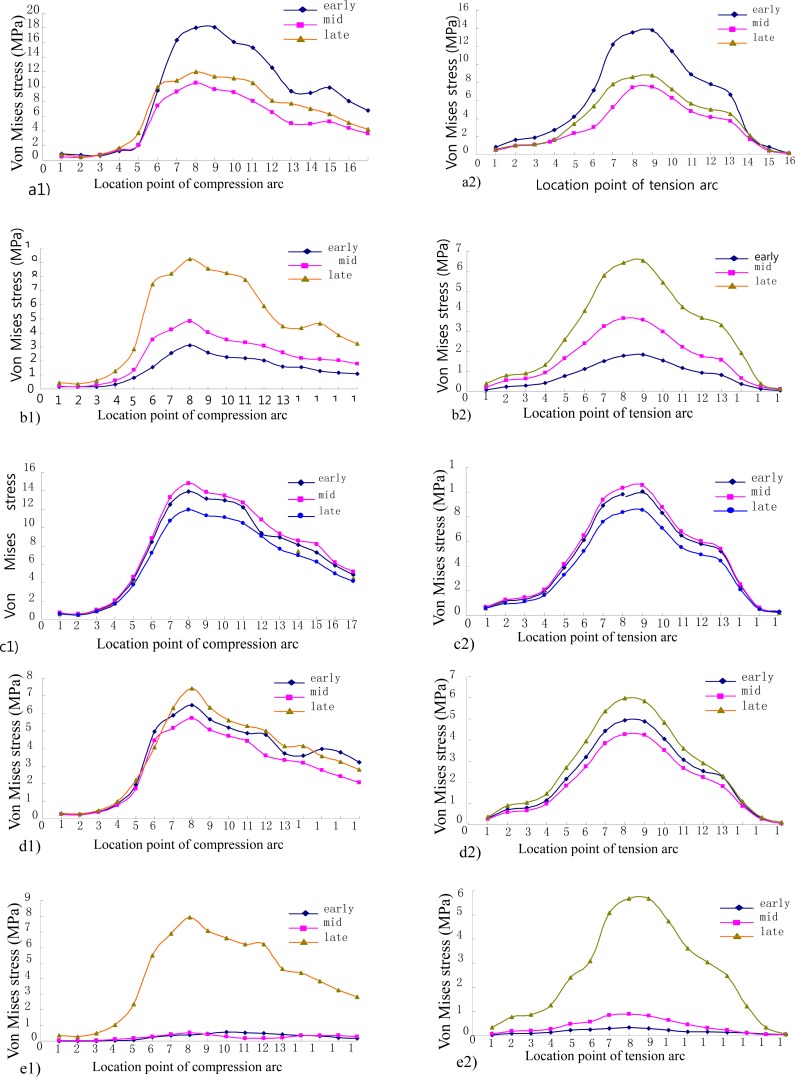
Compression (a1 – e1) and tension (a2 – e2) distribution across the femoral neck in the five tested fitness movements.

The stress distribution across the femoral head and neck wass demonstrated in Figure 5. When the femoral neck is under a bending moment, the inferior arc of the neck experiences the largest compressive stress while the superior arc observes the largest tensile stress. Along the tensile and compressive arches a total of 16 and 17 location points were identified respectively (Figure 6). Figure 6 shows the compressive and tensile stress curves interpolated across the location points across the femoral neck. For the compression and tension curves of the five movements, it can be seen that the maximum stresses are concentrated in the middle of the curves in most of the three phases. The location point 8 received the greatest compressive stress on the compression arc whereas the location point 9 experienced the greatest tensile stress on the tension arc (Figure 6). Table 2 summarized the peak stresses observed at the location points of the compression and tension arc and at the intersection point of the compressive and tensile stress lines of trabeculae (node 19949 - the center of the Ward’s triangle) during three phases of the five movements.

## Discussion

The purpose of the study was to analyze loading characteristics of femoral neck during five common fitness exercises. During the exercises, the arrangement of trabecular bones in proximal femur follows certain patterns. The longitudinal bone plates in the deep surface of the lesser trochanter, the femur arm, is the base of the trabecular bone inside of the weight-bearing system of the femoral neck. The internal compression trabecular system fans out to reach the femoral head, bears the compressive and tensile loading applied to the femoral head, and therefore serves as the internal stress bearing system of trabecular bones. For the external tension system of the cortical bones, the trabecular system reaches the outer arc and meets with the compression trabecular system at the femoral head. The two systems and the intertrochanteric crest intersect to form the central triangular region of the femoral neck, Ward’s triangle, where the structures are typically weak (Figure 5). The trabecular structures are light weighted, optimally structured, and excellent in weight bearing, which are often compared to the weight structures such as arched street lights and crane. The upper cortical arc receives the largest tensile stress whereas the lower cortical arc receives the greatest compression under loading conditions (Figure 5). In addition, the Ward’s triangle is the intersection region of the compressive and tensile trabecular systems and the concentrated stress site for the cancellous bone within the femoral neck region, and is also the key area of osteoporosis of the femoral neck. In order to prevent fractures and increase bone mass, these regions should be of main interest (Rudman et al., 2006).

From the analysis of femoral stress distribution of the different gaits, the stress was concentrated on the location point 8 of the compression arc and location point 9 of the tension arc of the femoral neck. We also observed high tension stress at the Ward’s triangle region at the intersection region of the trabecular compression and tension stress lines. Therefore, this region was identified as the target area of interest. Because of complex geometry of the human femur, the stress concentration is produced at the femoral neck, especially at the compression and tension arcs. Clinically, the femoral neck is also prone to fracture.

Among the digitized location points along the compression and tension arcs, we found that the peak stresses were concentrated on the point 8 on the compression arc and the point 9 on the tension arc in the 3 movement phases of the five movements analyzed. The forward/backward lunge experienced the greater Von Mises stress for the compact bone of the femoral neck and followed by the lateral lunge, walking, single-leg standing and squat (Table 3).

The stress imposed on the spongy bone in the femoral neck (Ward’s triangle) during the five movements was different according to the calculations and the maximum stress occurred at the different time. For the walk, it occurred at heel strike of the supporting leg, the lowest squatting position in the squat, and the moment when the contralateral leg lifting off the ground in the single-leg standing and these moments were all in the early phases of the moments. However, the maximum stress occurred in the mid support when the center of gravity was at the lowest positions. The maximum stress reached during walking at the heel strike was much higher than the other movements and the stress was maintained at a relatively high level during the entire stance, reaching 59.79, 48.56 and 21.49MPa at the early, middle, terminal stance, respectively. For the spongy bones of the femoral neck, the order of the peak stress was different with the walking experienced the greatest stress and followed by the forward/backward lunge, bilateral lunge, single-leg standing and squat (Table 3).

Bone is a living tissue of a human and maintains a continuous growth pattern. Bone structure and mechanical properties are continuously modified to meet the demands of changes in external environments. Dynamic balance of bone modifications are not only influenced by age, endocrine and nutrition, but also affected by applied stresses (Estrada et al., 2002). According to the Wolff’s law and biomechanics point of view, stresses placed on bone and osteoblasts are modulated dynamically through an open-loop feedback system. When bone stresses increase or decrease, bone cells make continuous adjustments to the density and shape of the bone in order to adapt to changes in loading environments and promote bone deposition and/or resorption processes. The new osseous tissues formed through this process should show characteristics that are closer to “normal” and adaptable to the changed loading environments.

The compression and tension arcs of the femoral neck are the parts of the outer and compact bone layers, which experience bending moments and are the key structures against fracture. Our experimental results showed that high stresses were observed along both compression and tension arcs throughout the entire phase of the forward lunge. These results showed that the lunge gait can provide high and effective stress stimuli to the compact bone structures of the femoral neck and help achieve the goal of prevention of femoral neck fracture. On the other hand, the lateral lunge’s results showed that the stress on the superior and inferior arcs is not too high. However, the stresses of the superior and inferior arcs reach 56.74 and 79.16 MPa, respectively, during the terminal stance when the body mass was on the lunged leg. The stress level on the inferior arc was the highest and may post threats to the integrity of the femoral neck, especially in the high risk population.

In the Ward’s triangle, the bone mineral density in the patients with femoral neck fracture is shown to be lower than the normal population (Qian et al., 2009). The results of our simulations demonstrated that the center of the Ward’s triangle was always under a high stress state during the early, middle and terminal stance of walking. This is especially apparent at the time of heel strike during walking when the stress was the highest among all five examined fitness activities. Therefore, walking seems to offer an effective and simple method in improving bone mass and preventing osteoporosis.

Due to greater loading applied by athletes during their sport career, they may experience smaller risk of femoral neck fracture. Once they stop regular training, however, the bone density within the femoral neck region may decrease to the level of non-athletes due to removal of high loading. The risk of fracture for the older retired athletes would be higher compared to their younger athletic counterparts. To reduce the risk even further, they could perform the lunge and walking with weights to further stimulate osseous activity of the femoral neck region.

One of the limitations of the study is that only one subject was used and the subject’s age was 23 year old. The readers should be cautioned against applications of the results obtained from this case study to the elderly population. Applicability of the results to the elderly population who may suffer osteoporotic femoral fracture due to falls may be further limited due to the younger age of the subject. Future studies should focus on effects of the recommended exercises to the elderly population using a larger sample size.

In summary, we have successfully created the musculoskeletal model in LifeMOD and computed dynamic parameters, transformed the loading variables into the finite element model of the human femur, and calculated its stress distributions. The results showed that the highest stress was found at the location point 8 and 9 on the compression and tension arcs, respectively. The forward/backward lunge may provide greater stress to the superior tension arc and inferior compression arc to elicit greater and more effective stimulations to the structures and achieve increased formations of compact bones and bone mineral density. This exercise may prove to be an effective means for femoral fracture prevention. People with high risk of femoral fracture should avoid side-step movements. Finally, during the support phase in walking, the stress imposed on the Ward’s triangle was usually the highest, especially at the heel contact. Therefore, walking also seems to be an effective and simple method in improving bone mass and preventing osteoporosis as well as femoral neck fracture.

## Figures and Tables

**Table 1:** Hip joint contact forces in ANSYS of the early, mid, late stance phase of the five analyzed movements (N)

Phase	Direction	Walking	Squat	Standing	Forward Lunge	Lateral Lunge
Early	X	−436.256	33.721	−321.699	2035.245	−432.704
	Y	54.463	−49.68	1.907	−675.464	213.645
	Z	−659.772	137.319	−465.271	1282.975	816.452

Mid	X	−254.6	152.234	−338.354	1677.332	−740.884
	Y	46.466	−78.257	27.876	−517.963	208.021
	Z	−324.94	85.530	−495.364	1327.761	697.797

Late	X	−286.275	−209.728	−268.778	2704.73	2057.55
	Y	6.430	29.985	−2.484	−1041.66	−633.935
	Z	−393.877	−307.759	−408.378	920.814	2183.886

**Table 2:** Maximum stress of the location points during the five movements (MPa)

Phase	Walking		Squat		One-leg Standing		Forward Lunge		Lateral Lunge	

	Comp	Tension	compress	tension	compress	tension	compress	tension	compress	tension
Early	18.1	13.8	3.1	1.8	13.9	10.1	64.7	49.4	4.0	3.5
Mid	10.5	7.5	4.8	3.6	14.8	10.6	57.4	42.7	5.2	9.0
Late	12.1	8.8	9.3	6.6	11.9	8.6	74.4	59.9	79.2	56.7

Node 19949	59.8		4.7		17.1		27.8		25.2	

Comp – compression.

**Table 3:** The order of the maximum stress of compact and spongy bone in the five movements

	Order of Von Mises stress (maximum → minimum)
Compact	Forward lunge **>** lateral lunge **>** walking **>** single-leg standing **>** squat
Spongy	walking **>** forward lunge **>** lateral lunge**>** single-leg standing **>** squat
